# Exploring Emotional Violence in Intimate Relationships during Pregnancy in Central Tanzania: A Qualitative Descriptive Study

**DOI:** 10.24248/eahrj.v9i1.827

**Published:** 2025-09-30

**Authors:** Fabiola Vincent Moshi, Keiko Nakamura, Yuri Tashiro, Ayano Miyashita, Runa Katoh, Hideko Sato, Mayumi Ohnishi

**Affiliations:** a Department of Nursing Management and Education, School of Nursing and Public Health, the University of Dodoma, DODOMA, Tanzania; b Department of Global Health Entrepreneurship, Institute of Science, Tokyo, Japan; c Health Data Science Research Unit, Institute for Future Initiatives, The University of Tokyo, Japan; d Graduate School of Biomedical Sciences, Nagasaki University, Nagasaki, Japan

## Abstract

**Background:**

During pregnancy and the puerperal period, women become more sensitive both physically and emotionally, making them particularly vulnerable to Intimate Partner Violence (IPV). Despite this heightened vulnerability, emotional violence in intimate relationships during pregnancy remains under-researched. This study aimed to address this gap by exploring the experiences of emotional violence among pregnant women in central Tanzania.

**Method:**

This study employed a qualitative descriptive study design, utilizing purposive sampling to recruit twenty-nine (29) participants for both in-depth interviews (IDIs) and Focus Group Discussions (FGDs). The respondents were post-delivery mothers with infants aged 42 days to six months. Data analysis was conducted using an inductive thematic approach.

**Results:**

The women who participated in the study were, on average, 27.76 years old, with ages ranging from 19 to 43 years. Most of the women had attained only a primary level of education and resided in rural areas. Thematic analysis of their experiences revealed five key themes related to emotional violence during pregnancy: frequent conflict and arguments, verbal abuse and insults, emotional trauma resulting from persistent mistreatment, neglect and abandonment by partners, and coercive control marked by degrading and manipulative behavior.

**Conclusion:**

These findings highlight the multifaceted nature of emotional intimate partner violence and its profound impact on pregnant women's mental health. Addressing these issues requires comprehensive strategies that include raising awareness, enhancing support systems, and implementing targeted interventions to protect and empower women during this vulnerable period. Keywords: Emotional Violence, Intimate Partner, Experiences, Pregnancy, Tanzania.

## BACKGROUND

According to the World Health Organization (WHO), emotional violence encompasses behaviors that inflict emotional harm and erode a person's sense of self-worth or self-esteem.^1^ This form of violence includes, but is not limited to, constant criticism, threats, verbal abuse, and controlling or isolating behaviors. Emotional violence can be as damaging as physical violence and can have long-lasting effects on an individual's mental health and well-being.^2^

It is well-established that during the pregnancy-puerperal cycle, women become more sensitive both physically and emotionally, which makes them more vulnerable to Intimate Partner Violence (IPV).^2^ Studies also show that verbal violence during pregnancy is reported more frequently than other forms of IPV.^3^

The violence during pregnancy has two primary outcomes: fatal and non-fatal.^4^ Fatal outcomes encompass maternal homicides and suicides. On a global scale, maternal homicide rates range from 0.97 to 10.6 per 100,000 live births.^5^ The non-fatal outcomes of intimate partner violence include detrimental health behaviors such as alcohol and drug abuse, smoking during pregnancy, and delayed prenatal care.^4^. Additionally, IPV negatively impacts reproductive health, leading to low birth weight, preterm delivery, insufficient weight gain, obstetric complications, STIs/HIV, miscarriage, and unsafe abortion. Furthermore, IPV significantly affects physical and mental health, resulting in injuries, physical impairments, depression, difficulties or lack of attachment to the child, and adverse effects on the child's well-being.^4^

There is substantial evidence that women who have experienced IPV exhibit significantly lower utilization of available reproductive health services.^6^ IPV occurring during pregnancy severely impedes their access to antenatal care (ANC) and skilled birth attendance.^6^ Male partners often control household earnings, and a woman's ability to attend ANC and receive skilled birth attendance support hinges on her partner's cooperation. In abusive relationships, women are frequently denied this essential support, thereby obstructing their right to access these critical health services.

The IPV during pregnancy has severe consequences for maternal mental health. Research indicates that women experiencing IPV are nine times more likely to suffer from suicidal ideation during pregnancy.^7^ Additionally, those exposed to IPV are seventeen times more likely to experience depression.^8^ Evidence indicates that psychological violence is significantly associated with common mental disorders, even in the absence of other forms of violence.^9^ Furthermore, when psychological violence is combined with physical or sexual violence, the risk of common mental disorders increases substantially.^9^

A previous study conducted in the Lake Zone of Tanzania revealed that the prevalence of depressive and anxiety symptoms among women was 25.39% and 37.31%, respectively, underscoring the significant burden of maternal mental health challenges in the region.^10^ The study further highlighted that strong partner support was associated with decreased odds of developing these conditions, emphasizing the vital role of supportive intimate relationships during pregnancy and the postpartum period in promoting women's mental well-being.^10^ Although the prevalence of intimate partner violence (IPV) in Tanzania has historically been highest in the Lake Zone (47.6%), the Central Zone follows closely, with a prevalence of 44.4%, making it the second most affected region.^11^ The risk of IPV in the Central Zone may be heightened by its status as the nation's capital and its role as a major destination for internal migration. As individuals from various parts of the country bring diverse cultural norms and gender dynamics, the region is rapidly transforming into a complex socio-cultural landscape. This evolving context makes it essential to explore the lived experiences of emotional violence during pregnancy, as shifting relationship dynamics and emerging stressors may uniquely influence the patterns and impact of IPV in this setting.

Therefore, the study aimed to delve into the often-overlooked emotional violence experienced by pregnant women in intimate relationships. By exploring these silent struggles, the study would illuminate the profound impact of such violence on maternal mental health.

## METHODS

### Study setting

The study was conducted in Dodoma and Singida, the two major regions of Central Tanzania, including three districts from each region. In Dodoma Region, the selected districts were Dodoma City, Kondoa District, and Mpwapwa District, while in Singida Region, the study covered Manyoni District, Ikungi District, and Singida Municipal Council. District health facilities within these six districts served as meeting points for engaging with postnatal women who met the inclusion criteria. Central Tanzania is made up of three regions (Dodoma, Singida and Manyara). As of the latest estimates, Central Tanzania has a population of approximately seven (7) million people, among them 3.52 million are female.^12^

Women of reproductive age (15–49 years) constitute a substantial segment of the population. The fertility rate in Central Tanzania (3.9 in Dodoma, 3.8 Singida and 2.8 Manyara) is relatively high, reflecting broader trends in rural Tanzania.^15^ This high fertility rate is influenced by cultural, socio-economic, and educational factors and has significant implications for healthcare services, education, and economic development.

### Study Design

A descriptive qualitative study was conducted to explore women's experiences of emotional violence during pregnancy.

### Study Population

The study included postnatal mothers, ranging from 42 days to six months postpartum, attending postnatal clinics in six selected health facilities in Central Tanzania. The inclusion criteria specified that participants must be postnatal mothers who had recently given birth (within 42 days to six months), had experienced intimate partner violence during their most recent pregnancy, were living with a male partner during this period, and had consented to participate in the study. The exclusion criteria ruled out postnatal mothers who, despite meeting the inclusion criteria, were seriously ill or had seriously ill infants at the time of data collection.

### Sample Size

Previous studies indicate that ten to thirty in-depth interviews (IDIs) results in data saturation, which is facilitated by the aim of the study, sample capacity, and quality of dialog.^13^ Consequently, this study commenced with ten participants for the in-depth interviews and continued recruiting until saturation was reached with the 19^th^ participant. For the Focus Group Discussions (FGDs), prior research suggests that four to eight FGD sessions with six to twelve participants per session can achieve data saturation, particularly among homogenous groups.^14^ Accordingly, this study conducted five FGDs with a total of twenty-nine participants. Of these, seventeen participants took part in both the IDIs and FGDs, two participated only in the IDIs, and ten participated solely in the FGDs.

A total of 29 women participated in this study. Of these, 17 participated in both in-depth interviews (IDIs) and focus group discussions (FGDs), while 2 participated only in IDIs and 10 only in FGDs.

### Sampling Technique

A purposive sampling technique was employed to recruit participants who had experienced intimate partner violence (IPV) during pregnancy, using an adapted version of the Hurt, Insult, Threaten, and Scream (HITS) screening tool. The extended HITS (E-HITS) tool comprises five items that assess the frequency of abusive behaviors experienced during pregnancy. Specifically, participants were asked: (1) During pregnancy, how often did your partner physically hurt you? (2) Insult or talk down to you? (3) Threaten you with harm? (4) Scream or curse at you? and (5) Force you to engage in sexual activities? Responses were recorded on a five-point Likert scale ranging from 1 (never) to 5 (frequently), yielding a total score between 5 and 25. Women who scored above 7 were classified as experiencing IPV during pregnancy and were included in the study.

### Data Collection Methods

The IDI and the FGD were the primary data collection methods. The overlapping participation was intentional and allowed for a more nuanced understanding of the topic, enabling triangulation of perspectives across different data collection methods.^15^ In the analysis, each data source IDI and FGD was treated independently to ensure that individual voices were not overrepresented. Data saturation was considered achieved when no new themes emerged during subsequent interviews and discussions.

Twelve research assistants participated in the study, six were nursing faculty members holding a Bachelor of Science in Nursing, while the remaining six were practicing nurses with diploma-level qualifications. The Principal Investigator conducted a one-day training session for all research assistants, focusing on the study tools, interview procedures, handling emotionally sensitive topics, and recognizing signs of participant distress. All research assistants were fluent in Kiswahili and experienced in counseling. The interview guides were pilot tested with a small group of postnatal mothers (n=6) from a health facility, which was not selected for actual data collection. The pilot aimed to assess the clarity, cultural appropriateness, and sequencing of questions. Feedback from the pilot led to minor modifications, including rephrasing of certain questions and adjusting the flow to improve comprehension and engagement during interviews.

### Data Collection Proceduer

#### In-depth Interviews

The data collection procedure was conducted following the training of the research assistants. To minimize distractions, research assistants set aside their phones during the interviews. The IDI interviews were conducted in a private room within the health facility. The researcher sat at an appropriate distance (30–39 inches) or at a 90-degree angle to the interviewee to facilitate comfortable interaction, and the interviews were conducted in Kiswahili.

Each IDI interview began with the researcher briefing the participant about the interview process and addressing any queries before starting. The interview started with a neutral question, followed by the key questions of the study. Each question was asked individually, allowing sufficient time for the participant to respond. Probing questions were employed when further exploration of an idea was needed based on the participant's responses. The interviews lasted approximately 45 to 60 minutes and were audio-recorded with the participant's consent. IDIs were conducted to explore participants’ personal and sensitive experiences of IPV during pregnancy in a private and confidential setting. This method enabled the collection of rich, individualized narratives, revealing the nuanced physical and emotional impacts of IPV.

### Focus Group Discussions

The focus group discussion (FGD) was facilitated by two researchers: a moderator and a note-taker. Women gathered in a designated room within the health facility, arranged to face each other to foster a sense of equality and minimize hierarchy among the group members. The lead researcher began by briefing the participants about the discussion, allowing time for introductions, establishing ground rules, and addressing any queries from the participants.

The researcher then posed open-ended questions, as outlined in the interview guide, to elicit in-depth responses. Follow-up questions were used as needed to seek clarification. Each FGD session, comprising 6–12 participants, lasted between 60 and 90 minutes, which is considered a standard duration to gather comprehensive information.^16^ The discussions were audio-recorded with the formal consent of all participants. FGDs were conducted to explore community-level perceptions, social norms, and shared attitudes toward IPV during pregnancy. This method captured collective experiences and societal context through group interactions, revealing common themes that may not surface in individual interviews.

### Data Collection Tools

Two data collection tools were employed: an In-Depth Interview Guide and a Focus Group Discussion Guide. The In-Depth Interview Guide comprised sections on the sociodemographic characteristics of respondents and their experiences of emotional violence in intimate relationships during pregnancy. Similarly, the Focus Group Discussion Guide included questions on the sociodemographic characteristics of respondents and their experiences of emotional violence in intimate relationships during pregnancy.

### Data Analysis and Presentation

Data from IDIs and FGDs were initially reviewed separately to capture the unique insights offered by each method. This approach enabled a more nuanced understanding of participants’ perspectives across different settings. Following transcription and familiarization with the data, initial codes were generated inductively from the content. These codes were then organized into potential sub-themes and overarching themes through an iterative, collaborative process involving multiple team members. To enhance reliability, two researchers independently coded a subset of transcripts and resolved discrepancies through discussion and consensus. Themes were then compared and integrated across IDIs and FGDs to strengthen the overall findings and ensure methodological triangulation. Representative quotes were used to illustrate each theme, and thick descriptions were provided to preserve context and convey deeper meaning.

### Rigor

Each transcription was initially analyzed by the first author, who identified the preliminary codes. These transcripts were then reviewed by the remaining members of the research team. The first and second authors consolidated similar codes. Subsequently, two authors convened in a face-to-face meeting to discuss the codes and agree on the key themes, thereby validating the accuracy of the interpretations and enhancing the study's rigor.

### Researcher Reflexivity

The research team consisted of individuals with backgrounds in public health, nursing, and qualitative research, some of whom had prior experience working in maternal and reproductive health settings in Tanzania. The lead researcher is a female academic and public health nurse with experience in intimate partner violence research, which informed both the study design and the sensitivity applied during data collection. This positionality may have influenced participants’ comfort in disclosing intimate experiences, particularly among women. To minimize bias, we maintained a reflective journal throughout the study, engaged in regular peer debriefings, and incorporated team discussions to challenge assumptions and interpretations. These strategies were employed to enhance the credibility and trustworthiness of the findings.

### Reporting Method

We have adhered to the Consolidated Criteria for Reporting Qualitative Research (COREQ) checklist.

### Ethics Approval and Consent to Participate

The study obtained ethical approval from the University of Dodoma's Institutional Research Review Committee (Ref. No. MA.84/261/02) and received permission from the Regional Administrative Officers of Dodoma and Singida Regions. Given the sensitive nature of the study, additional precautions were taken to ensure the emotional and psychological safety of participants. All participants were informed, both verbally and in writing, about the study's objectives, procedures, potential risks (including emotional distress), and expected benefits. Informed consent was obtained in both verbal and written form.

Interviews were conducted in private, safe locations chosen by the participants to ensure confidentiality and comfort. All research assistants were trained in handling emotionally sensitive topics and recognizing signs of participant distress. Participants were reminded of their right to pause or withdraw from the interview at any time without providing a reason or facing any consequences. In cases where participants showed signs of distress, immediate emotional support was offered, and referrals were provided to local counseling services.

To maintain anonymity, participants were assigned unique identification codes, and no personal identifiers were recorded in transcripts or data files. All digital and physical data were securely stored under password-protected and locked conditions accessible only to the principal investigator.

## RESULTS

### Socio-demographic Characteristics

The mean age of the respondents was 27.76 ± 7.074 years, with a range from 19 to 43 years. Their male partners had a mean age of 32.97 ± 10.192 years, ranging from 22 to 65 years. The majority of the interviewed women had a primary level of education, resided in rural areas (Table 1).

**TABLE 1:** Socio-demographic Characteristics of Study Participants

Variable	Frequency (n)	Percent (%)
Region of a participant		
Dodoma	11	37.9
Singida	18	62.1
Education level of respondents		
No formal Education	2	6.9
Primary Education	15	51.7
Secondary Education	9	31
College or University Education	3	10.3
Education level of a partner		
No formal Education	1	3.4
Primary Education	17	58.6
Secondary Education	7	24.1
College or University Education	4	13.8
Religion		
Christian	13	44.8
Muslim	16	55.2
Residence		
Rural	19	65.5
Urban	10	34.5

### Study Themes

**FIGURE 1:**
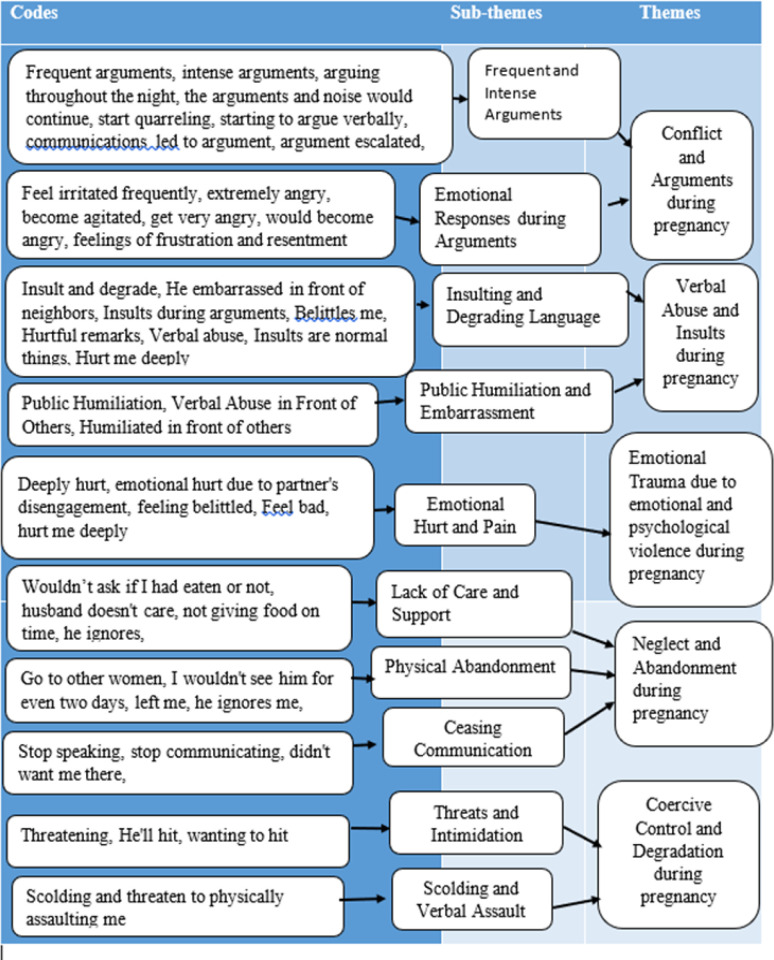
Emerged Themes and Sub-themes

Five primary themes emerged from the inductive thematic analysis network, as illustrated in Figure 1. These themes are 1) Conflict and Arguments during pregnancy, 2) Verbal Abuse and Insults during pregnancy, 3) Emotional Trauma Resulting from Emotional Violence during pregnancy, 4) Neglect and Abandonment during pregnancy, and 5) Coercive Control and Degradation during pregnancy. Notably, all these themes organically surfaced from the analysis and were not predetermined.

### Conflict and Arguments During Pregnancy

Women in both IDIs and FGDs explained that conflicts and arguments when they were pregnant posed a significant emotional abuse. Two sub-themes emerged from the analysis: Escalation of Verbal Conflicts and Emotional Responses during Arguments.

Participants vividly described the escalation of verbal conflicts during pregnancy, emphasizing how these arguments were a constant presence throughout their pregnancies. This pervasive nature of conflict and argument during pregnancy was underscored by a respondent from Kondoa district, who shared her frequent experiences of conflict and argument throughout this period.

“*I experienced Intimate Partner Violence from my partner, marked by frequent arguments and constant misunderstandings during my pregnancy*.”

(28 years old, Kondoa, IDI).

Another respondent also shared her experience of constant arguments during her pregnancy.

“*Each time he left and came back, the arguments and shouting would start again and never seemed to end*.”

(30 years old, Kondoa, IDI).

The reasons for arguments were often unclear; respondents found themselves arguing without being able to identify the underlying causes. Suspicion of a pregnant woman having an affair with other men was enough to trigger an argument, as confirmed by another respondent from Kondoa district.

“*When I was pregnant, he argued with me and insulted me without any clear reason. At times, he falsely accused me of being with other men and started quarrels over it*”

(20 years old, Kondoa, IDI).

Furthermore, the conflict and arguments during pregnancy were sometimes caused by a small misunderstanding between intimate couples. A respondent from Ikungi District mentioned that when she was pregnant even a minor issue often escalated into arguments.

“*Sometimes, even over something minor, we would argue, and he would spend the entire day cursing at me or throwing insults*,”

(23 years old, Ikungi, IDI).

Pregnant women expected protection and support from their intimate partners during this crucial period. However, contrary to their hopes, these partners often became the source of disharmony. A participant from Manyoni District highlighted that the man responsible for the pregnancy frequently prompted conflict, exacerbating the situation

“*At times during pregnancy, the man who impregnated you becomes the source of constant arguments*,”

(20 years old, Manyoni, FGD).

In the second sub-theme, interviewed women reported experiencing emotional responses such as irritation, extreme anger, agitation, frustration, and resentment as common outcomes during conflicts and arguments while pregnant. This was surfaced by a respondent from Ikungi district.

“*It's common to feel more easily irritated during pregnancy, as emotions tend to be heightened and sensitivity increases*”

(39 years old, Ikungi, IDI).

Another woman from Kondoa explained that the pregnancy period is associated with emotional vulnerability.

“*Challenges are inevitable, especially during pregnancy when you may not feel your best. At times, heightened emotions can lead to irritability, making even minor actions by your partner feel like major mistakes*.”

(28 years old, Kondoa, IDI).

Participants openly shared their experiences of these profound emotional responses throughout their pregnancies, highlighting the significant psychological impact of such discord. A respondent from Kondoa District detailed her experience of emotional abuse from her intimate partner during pregnancy, which triggered intense emotional reactions.

“*During pregnancy, intense anger in arguments sometimes caused me to lose consciousness*,”

(28 years old, Kondoa, IDI).

### Verbal Abuse and Insults during Pregnancy

Within this theme, participants reported experiencing constant verbal abuse and insults from their male partners during pregnancy. Two sub-themes emerged: the use of insulting and degrading language, and public humiliation and embarrassment. In the first sub-theme, participants explained that their intimate partners used degrading language to insult them. A respondent from Mpwapwa shared that when she was pregnant, her intimate partner would insult her with hurtful remarks.

“*The cruelty I experienced from my husband during pregnancy included insults, hurtful remarks, verbal abuse, and, at times, being denied money for basic needs*”

(27 years old, Mpwapwa, IDI).

Another respondent from Kondoa also shared that her intimate partner constantly argued with her during pregnancy, and these arguments often involved insulting words.

“*During my pregnancy, he frequently initiated arguments, about four times in total, and each time, the arguments quickly escalated into insults and verbal abuse*,”

(34 years old, Kondoa, IDI).

Additionally, another woman shared her experience during pregnancy in the in-depth interview, mentioning that insults often stemmed from her position as a woman, particularly when she requested financial support.

“*When I asked him for money, he insulted me with contempt. His belittling words hurt me deeply. Why did he treat me that way?*”

(24 years old, Kondoa, IDI).

The second sub-theme was public humiliation and embarrassment during pregnancy. Participants reported that insults were often delivered in front of others, leading to significant humiliation and embarrassment. Several women shared their experiences of public humiliation while pregnant. For instance, a woman from Kondoa recounted how her intimate partner threw her clothes out, declaring he no longer wanted her, despite her being pregnant.

“*He threw my clothes out and embarrassed me in front of the neighbors*,”

(20 years old, Kondoa, IDI).

Additionally, another woman in the focus group discussion said that a pregnant woman to be humiliated in-front of friends or relatives do happen frequently

“*Insults and humiliation in public happen often. A man may confront and shame a pregnant woman anywhere, whether she's with friends or family, without waiting to speak in private*,”

(43 years old, Ikungi, FGD)

Furthermore, another woman from Ikungi District reported an incident where her friend experienced verbal abuse from her intimate partner in front of their children.

“*Despite being pregnant and often ill, I struggled with my partner to provide for the family, but he spent all the money and insulted me*,”

(23 years old, Ikungi, FGD).

It was also noted that, due to these embarrassments, some women felt disrespected by those around them and preferred to walk outside with their faces covered.

“*When someone insults you deeply, it can make you feel so bad that you wish you could hide your face*”,

(23 years old, Manyoni, IDI).

### Emotional Trauma due to Emotional Violence during Pregnancy

Through the interviews, women expressed their profound emotional trauma stemming from emotional violence in their intimate relationships. This violence significantly undermined their well-being during the crucial period of pregnancy. One sub-theme that emerged within this context was emotional hurt and pain.

Participants reported that conflicts, arguments, insults, and humiliation inflicted profound emotional distress and anguish. One participant from Kondoa vividly recounted how her partner's behavior, including physical abuse and frequent arguments, deeply wounded her.

“*We often fought until we stopped speaking for weeks. When he came home, we ignored ` each other, and it deeply hurt me*,”

(28 years old, Kondoa, IDI).

Additionally, some male partners expressed regret over their female partners’ pregnancies. Living with an intimate partner who denies responsibility for caring for a pregnant woman is deeply distressing. Denying responsibility for the pregnancy is a clear indication of avoiding one's responsibilities. This sentiment was solidified by a woman in an in-depth interview in Mpwapwa district.

“*During my pregnancy, most abuse was through hurtful and disrespectful words. He would say things like, ‘I don't know why you even kept the pregnancy; you should have terminated it*,”

(34 years old, Mpwapwa, IDI).

Furthermore, another participant from Manyoni District stated that the maltreatment she endured from her male partner caused her severe emotional suffering.

“*The way he treated me when I was pregnant hurt me deeply. When someone leaves you in a state of pregnancy, the challenges I went through were immense and not insignificant*,”

(38 years old, Manyoni, IDI.

During interviews, women conveyed that their intimate partners disregarded them when they communicated feeling unwell. They emphasized that this behavior caused significant emotional trauma during pregnancy. This sentiment was shared by a woman from Ikungi district.

“*During pregnancy, one of the hardest experiences was my partner's indifference to how I felt. It seemed he didn't care, even when I told him I was unwell.”*,”

(43 years old, Ikungi, IDI)

### Neglect and Abandonment during Pregnancy

In this theme, two sub-themes emerged: physical abandonment during pregnancy and cessation of communication during pregnancy. Some male partners abandoned their women during pregnancy, either by moving away or starting new relationships. One respondent from Manyoni District shared her experience of physical abandonment when she was pregnant by her intimate partner.

“*When I was pregnant, he often neglected me. Sometimes he would leave to be with other women, and there were times I didn't see him for two days or more*

(36 years old, Manyoni, IDI).

Another participant in a focus group discussion mentioned that male partners often evade their responsibilities, leaving pregnant women to fend for themselves in securing meals. This occurs even though pregnancy significantly limits a woman's ability to earn a living.

“*These challenges affect us and are common among our neighbors and relatives. Often, a pregnant woman is neglected by her husband, left hungry while caring for small children*

(39 years old, Ikungi, FGD).

It was also reported that some male partners distanced themselves from providing financial support during pregnancy. A respondent from Kondoa shared that her partner would leave home without arranging for her meals, then return only to ask if she had eaten, before leaving again without offering any solutions.

“*During my pregnancy, my partner would leave and return to check on us, but never provided financial support. When I told him we lacked money, he simply said he had none*”

(20 years old, Kondoa, IDI).

In the second sub-theme, respondents reported periods during pregnancy when communication ceased entirely, despite living together. A respondent from Kondoa described instances where they didn't speak to each other for two weeks. She emphasized that living together in complete silence was a particularly difficult and distressing experience for her.

“*We stopped speaking for weeks. When he came home, we each kept to ourselves, but I was deeply hurt by the distance between us*”

(28 years old, Kondoa, IDI).

Another respondent from Manyoni mentioned that sometimes when she was pregnant, her male partner would choose to remain silent. In response, she would also stay silent, resulting in a situation where neither of them spoke to each other.

“*Sometimes he wouldn't respond and just stay silent; other times, he became angry. So, I decided to ignore him as well*.

(36 years old, Manyoni, IDI)”

On the other hand, some women decided to keep quiet to avoid the consequences of arguments with their male partners.

“*During pregnancy, I chose to avoid communicating with him to prevent arguments, although he still provided financial support*,”

(34 years old, Mpwapwa, IDI).

### Coercive Control and Degradation during Pregnancy

The interviews revealed that male partners exerted control over their female partners through threats and harsh reprimands during pregnancy. Two prominent sub-themes emerged from this overarching theme: threat and intimidation during pregnancy, and scolding and verbal assault during pregnancy.

Women faced severe threats and harsh reprimands from their intimate partners. A woman from Ikungi district reported that her male partner frequently threatened her, particularly when he wanted to have sexual intercourse and she did not consent.

“*During pregnancy, threats and forced sex occurred repeatedly, with him insisting and threatening to hit me if I refused*”

(23 years old, Ikungi, IDI).

Another woman from the Ikungi district stated that sometimes the threats occur following arguments.

“*At times, he would return and immediately start insults, sometimes threatening to kick me out or even hit me*

(23 years old, Ikungi, IDI)

It was also revealed during the interview that some women were scolded and verbally assaulted by their male partners.

“*If I was late, he accused me of being with other men and started quarrelling. He insulted and degraded me in every possible way*”

(20 years old, Kondoa, IDI).

## DISCUSSION

Emotional violence during pregnancy is a critical concern that significantly impacts women's well-being. This study aimed to understand women's experiences of emotional violence in intimate relationships during pregnancy. The findings revealed that pregnant women experienced various forms of emotional violence characterized by persistent conflict, psychological distress, and a lack of support from their partners. These experiences often involved controlling behaviors, verbal abuse, and feelings of abandonment, which significantly affected their emotional well-being. The results highlight the complex and deeply rooted nature of emotional abuse during pregnancy.

The findings should be understood within the cultural context of Central Tanzania, where patriarchal norms strongly influence gender roles and power dynamics in intimate relationships.^17^ In this setting, men are often viewed as the primary decision-makers, and women, especially pregnant women, may face social pressure to tolerate mistreatment to preserve family unity or avoid community stigma.^18^ Cultural expectations around silence, obedience, and the normalization of verbal and emotional abuse can further inhibit help-seeking behavior, reinforcing the cycle of violence

Conflict and arguments during pregnancy signify a major communication breakdown during this crucial period. In this study, women reported experiencing frequent and intense conflicts in their intimate relationships during pregnancy. Similar studies have reported that conflict and arguments persist in intimate relationships during pregnancy.^19, 20^ It is also evident that pregnancy is a particularly vulnerable period for such conflicts and arguments due to the emotional changes caused by pregnancy.^21^

Communication breakdown during this critical period has severe consequences for the family. Expectant families are expected to plan for birth and prepare for unexpected emergencies, including attending antenatal clinics, recognizing danger signs, and gathering necessary birth items. Successful birth preparedness and complication readiness hinge on the collective efforts of expecting parents. A previous study conducted in Tanzania reported that poor household communication has led to non-facility childbirth assisted by unskilled birth attendants, underscoring the vital importance of effective communication during pregnancy.^22^

In a male-dominant country like Tanzania, support from intimate partners is crucial for the mental and physical health of pregnant women. Male partners typically control household earnings and are responsible for ensuring their partners attend health checkups and access nutritious food throughout the perinatal period.^23^ In situations of conflict and argument, women are often unjustly denied the essential care and support they deserve during pregnancy.

Male partners’ understanding of the emotional changes during pregnancy can significantly alleviate conflict and argument situations. Investing in the empowerment of male partners, educating them on how to cope with and support their pregnant spouses through these emotional changes, can greatly reduce conflicts and foster a more supportive and harmonious environment during pregnancy.

The second dimension of emotional violence identified in the study was verbal abuse and insults. In this context, women shared experiences of their male partners using insulting and degrading language. This behavior may be influenced by women's subordinate position in society, where male partners often dominate and may perceive their female partners as property. Such a perception can lead to a profound loss of respect for their intimate partners. It is evidenced that during pregnancy, the situation of insults can be aggravated, particularly when women are highly dependent on their intimate partners for their basic needs.^24^ This dependency often arises because many women lose their ability to contribute to the family income during this period. For those in informal employment, this can mean losing their jobs due to decreased productivity.

Additionally, women in this study recounted situations where they were publicly humiliated and embarrassed by their intimate partners during pregnancy. Public humiliation can be a means for the male partner to exert control and power, reinforcing dominance and subordination. Studies have shown that male partners might humiliate their spouses publicly due to feelings of insecurity or jealousy, attempting to undermine the spouse's confidence and self-esteem to feel more powerful or secure. ^24^

Furthermore, women reported episodes of emotional trauma stemming from their intimate partners’ abusive treatment, which led to prolonged periods of hurt and pain. Such sustained emotional abuse can result in severe mental health issues, including depression, anxiety, and Post-Traumatic Stress Disorder (PTSD). Additionally, victims of emotional violence may exhibit behavioral changes, often turning to substance abuse, such as drugs and alcohol, as coping mechanisms. Expressions of hurt and pain are clear indicators of the mental damage experienced during pregnancy, highlighting the profound psychological impact of emotional violence during this vulnerable period.

According to this study, the fourth dimension of emotional violence was male partners’ neglect and abandonment of their pregnant spouses. In this dimension, pregnant women were either physically abandoned or experienced a cessation of communication. Experiencing neglect and abandonment from an intimate partner is a significant emotional trauma. Some women were abandoned while their partners lived with other women, and some did not even know where their partners were. A previous study conducted in Vietnam reported similar findings.^20^ The study recommends serious measures to be taken against male partners who avoid their responsibilities and subject their pregnant spouses to complications related to pregnancy and childbirth. It is crucial to hold these individuals accountable to ensure the health and well-being of both the mother and the unborn child.

The study also revealed that women frequently experience coercive control and degradation within their intimate relationships during pregnancy. This form of emotional violence assumes heightened significance during pregnancy due to shifts in relationship dynamics, where the pregnant partner often requires increased support and attention. Some men, feeling a loss of control, may resort to coercive tactics to regain dominance. Moreover, the impending responsibilities of fatherhood can trigger considerable stress and anxiety, leading some men to manifest these emotions through controlling and degrading behaviors directed at their partners.^25^ This exposes women to coercive control and degradation during pregnancy, which profoundly affects their mental health.

The study underscores the importance of the healthcare system offering counseling services that address healthy relationship dynamics, stress management, and the emotional challenges specific to pregnancy.

The findings highlight an urgent need for healthcare providers to be trained in identifying and responding to signs of intimate partner violence during pregnancy. Routine screening for IPV in antenatal care settings, along with referral pathways to counseling and social support, could play a critical role in early intervention. For policymakers, the study emphasizes the importance of strengthening legal protections for pregnant women, supporting community-based awareness campaigns to challenge harmful gender norms, and integrating IPV prevention into national reproductive health strategies. Context-specific and culturally informed policies will be key to addressing the unique challenges faced by women in Central Tanzania

While the study employed a qualitative descriptive design, which emphasizes straightforward descriptions over theoretical interpretation or deep causal explanation, it nonetheless ensured rigor through systematic data collection and thematic analysis. The use of IDIs and FGDs, triangulated across multiple districts and participant backgrounds, provided rich, contextually grounded insights. Additionally, adherence to established qualitative rigor criteria (credibility, dependability, confirmability, and transferability) strengthened the trustworthiness of the findings. The study may also be subject to social desirability bias, as participants might have underreported or downplayed their experiences of violence due to stigma or fear of judgment. Recall bias may also have affected the accuracy of participants’ accounts, especially in recalling emotionally charged events. The study's findings are context-specific to Central Tanzania and may not be generalizable to other regions. Furthermore, although interviewers were trained and experienced in counseling, their presence and approach may have influenced participants’ responses during interviews and focus group discussions.

## CONCLUSION

The study found that women experienced emotional violence during pregnancy in multiple, interrelated forms that severely affected their mental and emotional well-being. These included patterns of disrespect, psychological manipulation, and lack of partner support that created a persistent sense of fear, distress, and isolation. These findings underscore the multifaceted nature of emotional intimate partner violence and its profound impact on pregnant women's mental health. Addressing these issues requires comprehensive strategies, including raising awareness among male partners about the emotional changes during pregnancy, enhancing support systems by assessing emotional violence during antenatal visits, and implementing targeted interventions to protect and empower women during this vulnerable period. By shedding light on these critical issues, this study aims to inform future research, policy development, and clinical practice to improve maternal health outcomes in Tanzania and beyond.
